# DISEASE-SPECIFIC CONTRIBUTIONS TO GEOGRAPHICAL DISPARITIES IN MORTALITY AMONG MEDICARE BENEFICIARIES

**DOI:** 10.1093/geroni/igae098.1213

**Published:** 2024-12-31

**Authors:** Meishuo Ouyang, Igor Akushevich, Julia Kravchenko

**Affiliations:** Duke University School of Medicine, Durham, North Carolina, United States; Duke University, Durham, North Carolina, United States; Duke University School of Medicine, Durham, North Carolina, United States

## Abstract

Improving Life expectancy (LE) in the LE-lagging states could significantly improve overall LE and eliminate health inequalities in the United States. We analyzed the disparity in mortality rates between eight states with high and low LE at 65 using individual morbidity trajectories in Medicare administrative claims data from 2000-2020. Disease-specific contributions were identified using the Powers-Yun decomposition technique for hazard functions (the Blinder-Oaxaca algorithm extended for time-to-event data). This approach identifies two effects per disease: exposure (the disparity is generated because of the difference in the prevalence of a disease in two compared populations) and vulnerability (the disparity is generated because of the difference in mortality in the subpopulation with the disease). Two population groups (80-) and (80+) separated by age 80 were analyzed. Disease-specific contributions explained 44% and 63% of the disparities in all-cause mortality in the 80- and 80+ age groups, respectively. Exposure effects dominated in the 80- group (47.8% vs. 3.5% of vulnerability effect), while vulnerability was the main contributor (40.1% vs. 23.3% of exposure effect) in the 80+ group. Heart failure, influenza, and pneumonia explained 21.3% of the disparity in the 80- group, while the other circulatory system diseases, especially the vulnerability of cardiac dysrhythmias (5%), dominated the 80+ group. Additionally, cerebrovascular diseases (10.7%) and blood diseases (14.1%) are the other two major contributors to vulnerability in 80+. Reducing disparities in LE demands multiple fronts, such as tobacco control, heightened preventive measures against influenza and pneumonia, and enhanced access to arrhythmia treatments.

